# Brain reserve contributes to distinguishing preclinical Alzheimer’s stages 1 and 2

**DOI:** 10.1186/s13195-023-01187-9

**Published:** 2023-02-28

**Authors:** Zerrin Yildirim, Firuze Delen, David Berron, Hannah Baumeister, Gabriel Ziegler, Hartmut Schütze, Wenzel Glanz, Laura Dobisch, Oliver Peters, Silka Dawn Freiesleben, Luisa-Sophie Schneider, Josef Priller, Eike Jakob Spruth, Anja Schneider, Klaus Fliessbach, Jens Wiltfang, Björn-Hendrik Schott, Dix Meiberth, Katharina Buerger, Daniel Janowitz, Robert Perneczky, Boris-Stephan Rauchmann, Stefan Teipel, Ingo Kilimann, Christoph Laske, Matthias H. Munk, Annika Spottke, Nina Roy, Michael Heneka, Frederic Brosseron, Michael Wagner, Sandra Roeske, Alfredo Ramirez, Michael Ewers, Peter Dechent, Stefan Hetzer, Klaus Scheffler, Luca Kleineidam, Steffen Wolfsgruber, Renat Yakupov, Matthias Schmid, Moritz Berger, Hakan Gurvit, Frank Jessen, Emrah Duzel

**Affiliations:** 1 Department of Neuroscience, Aziz Sancar Institute of Experimental Medicine, Istanbul University, Vakif Gureba Cad., Capa Kampusu Sehremini, Fatih, 34093 Istanbul, Turkey; 2 Department of Neurology, Bagcilar Training and Research Hospital, University of Health Sciences, 34200 Istanbul, Turkey; 3 Department of Neurology, Basaksehir Cam and Sakura City Hospital, 34480 Istanbul, Turkey; 4 Institute of Cognitive Neurology and Dementia Research, Otto-von-Guericke University Magdeburg, Leipziger Str. 44, 39120 Magdeburg, Germany; 5 German Center for Neurodegenerative Diseases (DZNE), Magdeburg, Leipziger Str. 44, 39120 Magdeburg, Germany; 6 German Center for Neurodegenerative Diseases (DZNE), Berlin, Germany; 7 Charité – Universitätsmedizin Berlin, corporate member of Freie Universität Berlin and Humboldt-Universität zu Berlin-Institute of Psychiatry and Psychotherapy, Berlin, Germany; 8 Department of Psychiatry and Psychotherapy, Charité, Charitéplatz 1, 10117 Berlin, Germany; 9 Department of Psychiatry and Psychotherapy, School of Medicine, Technical University of Munich, Munich, Germany; 10 University of Edinburgh and UK DRI, Edinburgh, UK; 11 German Center for Neurodegenerative Diseases (DZNE), Venusberg-Campus 1, 53127 Bonn, Germany; 12 Department of Neurodegenerative Disease and Geriatric Psychiatry/Psychiatry, University of Bonn Medical Center, Venusberg-Campus 1, 53127 Bonn, Germany; 13 Department of Medical Sciences, Neurosciences and Signaling Group, Institute of Biomedicine (iBiMED), University of Aveiro, Aveiro, Portugal; 14 German Center for Neurodegenerative Diseases (DZNE), Goettingen, Germany; 15 Department of Psychiatry and Psychotherapy, University Medical Center Goettingen, University of Goettingen, Von-Siebold-Str. 5, 37075 Goettingen, Germany; 16 Leibniz Institute for Neurobiology, Magdeburg, Germany; 17 Department of Psychiatry, University of Cologne, Medical Faculty, Kerpener Strasse 62, 50924 Cologne, Germany; 18 German Center for Neurodegenerative Diseases (DZNE, Munich), Feodor-Lynen-Strasse 17, 81377 Munich, Germany; 19 Institute for Stroke and Dementia Research (ISD), University Hospital, LMU Munich, Feodor-Lynen-Strasse 17, 81377 Munich, Germany; 20 Department of Psychiatry and Psychotherapy, University Hospital, LMU Munich, Munich, Germany; 21 Munich Cluster for Systems Neurology (SyNergy) Munich, Munich, Germany; 22 Ageing Epidemiology Research Unit (AGE), School of Public Health, Imperial College London, London, UK; 23 Sheffield Institute for Translational Neuroscience (SITraN), University of Sheffield, Sheffield, UK; 24 Department of Neuroradiology, University Hospital LMU, Munich, Germany; 25 German Center for Neurodegenerative Diseases (DZNE), Rostock, Germany; 26 Department of Psychosomatic Medicine, Rostock University Medical Center, Gehlsheimer Str. 20, 18147 Rostock, Germany; 27 German Center for Neurodegenerative Diseases (DZNE), Tübingen, Germany; 28 Section for Dementia Research, Hertie Institute for Clinical Brain Research and Department of Psychiatry and Psychotherapy, University of Tübingen, Tübingen, Germany; 29 Department of Psychiatry and Psychotherapy, University of Tübingen, Tübingen, Germany; 30 Department of Neurology, University of Bonn, Venusberg-Campus 1, 53127 Bonn, Germany; 31 Excellence Cluster on Cellular Stress Responses in Aging-Associated Diseases (CECAD), University of Cologne, Joseph-Stelzmann-Strasse 26, 50931 Cologne, Germany; 32 Division of Neurogenetics and Molecular Psychiatry, Department of Psychiatry and Psychotherapy, Faculty of Medicine and University Hospital Cologne, University of Cologne, Cologne, Germany; 33 Department of Psychiatry & Glenn Biggs Institute for Alzheimer’s and Neurodegenerative Diseases, San Antonio, TX USA; 34 Department of Cognitive Neurology, MR-Research in Neurosciences, Georg-August-University Goettingen, Göttingen, Germany; 35 Berlin Center for Advanced Neuroimaging, Charité – Universitätsmedizin Berlin, Berlin, Germany; 36 Department for Biomedical Magnetic Resonance, University of Tübingen, 72076 Tübingen, Germany; 37 Institute for Medical Biometry Informatics, and Epidemiology, University Hospital Bonn, Venusberg-Campus 1, 53127 Bonn, Germany; 38 Department of Neurology, Behavioral Neurology and Movement Disorders Unit, Istanbul Faculty of Medicine, Istanbul University, 34093 Istanbul, Turkey; 39 Neuroimaging Unit, Istanbul University, Hulusi Behcet Life Sciences Research Center, 34093 Istanbul, Turkey

**Keywords:** Alzheimer’s disease (AD), Brain reserve, Subjective cognitive decline (SCD), Cerebrospinal fluid (CSF), Amyloid pathologic change, Aß42/40, Magnetic resonance imaging (MRI), Hippocampus, Medial temporal lobe, Memory

## Abstract

**Background:**

In preclinical Alzheimer’s disease, it is unclear why some individuals with amyloid pathologic change are asymptomatic (stage 1), whereas others experience subjective cognitive decline (SCD, stage 2). Here, we examined the association of stage 1 vs. stage 2 with structural brain reserve in memory-related brain regions.

**Methods:**

We tested whether the volumes of hippocampal subfields and parahippocampal regions were larger in individuals at stage 1 compared to asymptomatic amyloid-negative older adults (healthy controls, HCs). We also tested whether individuals with stage 2 would show the opposite pattern, namely smaller brain volumes than in amyloid-negative individuals with SCD. Participants with cerebrospinal fluid (CSF) biomarker data and bilateral volumetric MRI data from the observational, multi-centric DZNE-Longitudinal Cognitive Impairment and Dementia Study (DELCODE) study were included. The sample comprised 95 amyloid-negative and 26 amyloid-positive asymptomatic participants as well as 104 amyloid-negative and 47 amyloid-positive individuals with SCD. Volumes were based on high-resolution T2-weighted images and automatic segmentation with manual correction according to a recently established high-resolution segmentation protocol.

**Results:**

In asymptomatic individuals, brain volumes of hippocampal subfields and of the parahippocampal cortex were numerically larger in stage 1 compared to HCs, whereas the opposite was the case in individuals with SCD. MANOVAs with volumes as dependent data and age, sex, years of education, and DELCODE site as covariates showed a significant interaction between diagnosis (asymptomatic versus SCD) and amyloid status (Aß42/40 negative versus positive) for hippocampal subfields. Post hoc paired comparisons taking into account the same covariates showed that dentate gyrus and CA1 volumes in SCD were significantly smaller in amyloid-positive than negative individuals. In contrast, CA1 volumes were significantly (*p* = 0.014) larger in stage 1 compared with HCs.

**Conclusions:**

These data indicate that HCs and stages 1 and 2 do not correspond to linear brain volume reduction. Instead, stage 1 is associated with larger than expected volumes of hippocampal subfields in the face of amyloid pathology. This indicates a brain reserve mechanism in stage 1 that enables individuals with amyloid pathologic change to be cognitively normal and asymptomatic without subjective cognitive decline.

## Background

Alzheimer’s disease is characterized by a long preclinical course of amyloid and related pathologies before cognitive performance declines to the level of mild cognitive impairment (MCI). In this preclinical stage, the experience of progressive subjective cognitive decline (SCD) is considered a symptomatic indicator of stage 2 of the Alzheimer’s continuum, which is preceded by the asymptomatic stage 1 [1–3].

**Fig. 1:**
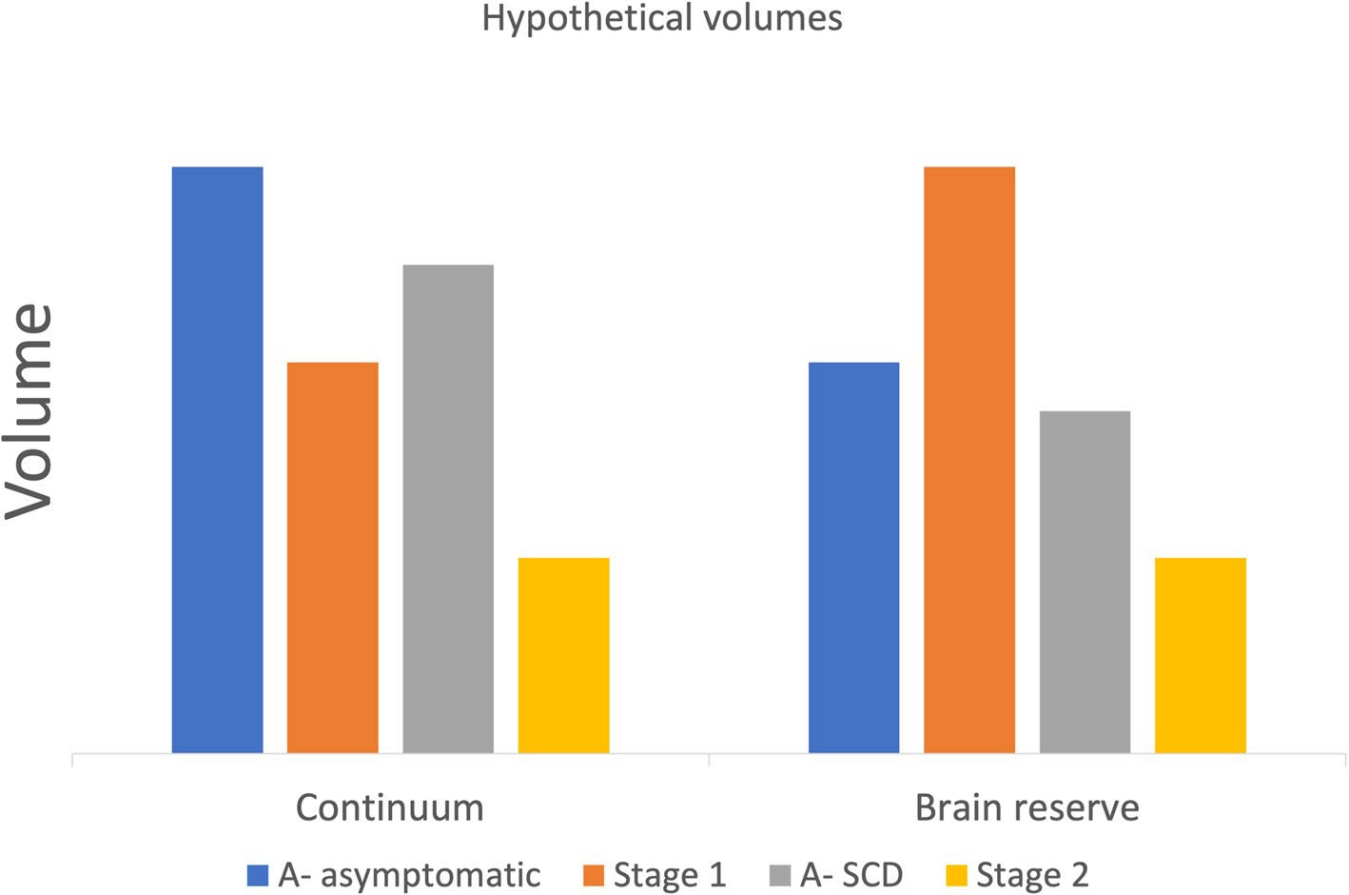
Hypothetical volumes of memory regions in amyloid-negative (A**−**) asymptomatic healthy controls, A**−** subjective cognitive decline (SCDs), stages 1 and 2. According to a continuum model, volumes should be gradually smaller from A**−** asymptomatic to 2. According to a brain reserve model, stage 1 should have higher volumes than A**−** asymptomatic (“higher than expected”), while stage 2 should show the expected decrease. The A**−** SCD group serves as a control that any volume differences between stages 1 and 2 are not solely attributable to the presence of memory complaints

An important question is why some individuals are asymptomatic and therefore fall into stage 1, while others experience symptoms and therefore fall into stage 2. One possibility is that both stages form a continuum of subtle preclinical progression with brain atrophy in which the degree of atrophy in stage 1 is not sufficiently severe to cause symptoms (Fig. 1). Alternatively, individual differences in brain reserve may impact on symptom manifestation in the presence of amyloid (Fig. 1). A commonly reported domain of subjective decline in the context of AD is related to episodic long term memory [1, 2, 4]. According to a continuous model, brain regions early affected in AD and related to memory function and subjectively perceived memory deficits would show evidence of subtle atrophy in stage 1 compared to a status without amyloid (Fig. 1). In stage 2, this atrophy would be more pronounced, causing subjectively experienced memory dysfunction. The alternative is the presence of intact brain reserve in stage 1, which would prevent subjectively experienced memory dysfunction and the absence of sufficient brain reserve at stage 2 (Fig. 1).

In the recent Reserve and Resilience working group research framework, brain reserve has been defined as better than expected brain integrity related to cognitive function in the face of a pathology [5]. According to this possibility, individuals in stage 1 may show larger volumes of memory-related brain regions compared to individuals without amyloid as an indicator of higher brain reserve. In stage 2, indicated by SCD, on the other hand, the reverse pattern could be expected and interpreted as an indicator of lower brain reserve (Fig. 1). In this scenario, the cross-sectional and longitudinal impact of amyloid pathology would depend on the presence of brain reserve. Individuals with low brain reserve would remain in stage 1 for a short duration and progress rapidly to stage 2, while those with high brain reserve would remain longer in stage 1 and progress slowly to stage 2.

The functional anatomical hallmark of episodic memory is the medial temporal lobe with the hippocampal formation and parahippocampal region [6–8]. In order to detect subtle atrophy patterns in these regions, it is worthwhile to assess the volumes of subfields in the hippocampal formation and subregions in the parahippocampal region (i.e., [8]). Recently, segmentation algorithms to detect the anatomical boundaries of subfields and subregions on MRI have considerably improved [9–11].

In this study, we measured the volumes of the hippocampal formation subfields, notably the dentate gyrus (DG), CA1, CA2/CA3, and subiculum according to a recently developed segmentation protocol for high-resolution T2 images [9]. We also measured volumes of parahippocampal regions, notably the entorhinal cortex (ErC), the perirhinal cortex with Brodmann areas 35 (transentorhinal cortex) and 36, and the parahippocampal cortex (PhC). We tested whether in asymptomatic individuals and those with SCD who were either amyloid-negative (A−) or amyloid-positive (A+), the volume pattern of the subfields and subregions was compatible with a continuum interpretation (amyloid negative > stage 1 > stage 2) or a brain reserve interpretation (stage 1 > amyloid negative; stage 1 > stage 2; interaction between amyloid status and the presence/absence of memory complaints). To that end, we utilized data from the multicentric DELCODE study of the German Center for Neurodegenerative Diseases.

We included the A− SCD group in the test of the continuum versus the brain reserve model, in order to account for the possibility that all patients with SCD have smaller hippocampi, irrespective of their amyloid status, when compared to asymptomatic individuals (resulting in a main effect of memory complaints). Such a possibility would argue against a role of amyloid pathology in explaining differences between stages 1 and 2. Therefore, the interaction of clinical stage (1 and 2) and amyloid status (A− and A+) with respect to brain volume would be a critical test of a brain reserve hypothesis (Fig. 1).

## Methods

The DELCODE study (for details, see [12]) is an observational longitudinal memory clinic-based multicenter (10 sites) study of the German Center for Neurodegenerative Diseases (DZNE) in Germany. It comprises the clinical stages of Alzheimer’s disease from stage 1 (asymptomatic and amyloid-positive) to stage 4 (early dementia) as well as amyloid-negative cognitive unimpaired individuals. Asymptomatic individuals were defined by an age-, sex-, and education-adjusted performance within − 1.5 standard deviations (SD) on all tests of the CERAD (consortium to establish a registry of Alzheimer’s disease test battery) cognitive test battery. SCD was defined by the presence of subjectively reported decline in cognitive functioning and a test performance above − 1.5 SD below the age-, sex-, and education-adjusted normal performance on all subtests of the CERAD [2]. Participants with SCD were referrals to the memory clinic including self-referrals while the control group was recruited by standardized public advertisement.

Additional inclusion criteria for both groups were age ≥ 60 years, fluent German language skills, capacity to provide informed consent, and presence of a study partner. For exclusion criteria, see [12].

The annual neuropsychological testing in DELCODE included the PACC5 [13]. The PACC5 *z*-score was calculated as the mean performance *z*-score across the Mini-Mental State Examination (MMSE), a 30-item composite screening test, the Wechsler Memory Scale Logical Memory Delayed Recall, a test of delayed (30 min) story recall, the Digit-Symbol Coding Test (DSCT; 0–93), a test of memory, executive function and processing speed, the Free and Cued Selective Reminding Test–Free Total Recall (FCSRT96; 0–96), a test of free and cued recall of newly learned associations, and the Category Fluency Test, a test of semantic memory and executive function [14–17]. The *z*-scores for the PACC5 in our analysis were derived using the mean and standard deviation of healthy controls and participants with SCD as well as relatives of patients with dementia in the entire DELCODE study.

All local institutional review boards and ethical committees approved the study protocol. All participants gave written informed consent before inclusion in the study. DELCODE is registered at the German Clinical Trials Register (DRKS00007966) (04/May/2015). Data handling and quality control are reported in [12].

### Sample

T1 and T2 MRI datasets were obtained from 916 participants at baseline. Of these, 433 participants had also CSF data available, which was used to define amyloid positivity (see below). The MRI data underwent automatic segmentation of hippocampal subfields and parahippocampal region according to the protocol outlined below. After manual inspection by an experienced rater (see below) 272 participants had hippocampal segmentations that passed quality assessment for both hemispheres. This final segmentation sample comprised 95 amyloid-negative (A−) asymptomatic individuals, 26 amyloid-positive (A+) asymptomatic individuals, 104 A− SCD, and 47 A+ SCD. The remaining participants of the segmentation sample had MCI and early dementia and were not considered for the current analysis.

### CSF Alzheimer’s disease biomarker assessment

CSF Alzheimer’s disease biomarkers were determined centrally at the Bonn site using commercially available kits according to vendor specifications (V-PLEX Aβ Peptide Panel 1 (6E10) Kit, K15200E and V-PLEX Human Total-tau Kit, K151LAE (Mesoscale Diagnostics LLC, Rockville, USA), and Innotest PhosphoTau (181P), 81581, Fujirebio Germany GmbH, Hannover, Germany) (also see [12]). Cut-offs were calculated from the DELCODE dataset by Gaussian mixture modeling using the R package flexmix (version 2.3-15). The following cut-offs were determined: Aß42 ≤ 638.7 pg/ml, Aß42/Aß40 ≤ 0.08 pg/ml, total Tau > 510.9 pg/ml, phospho-tau ≥ 73.65 pg/ml, and Aß42/phospho-tau < 9.68 pg/ml. The cut-off of the Aß42/Aß40 ratio was used to define amyloid positivity.

### MRI acquisition

MRI data were acquired at nine DELCODE scanning sites, all equipped with Siemens scanners (3 TIM Trio systems, 4 Verio systems, one Skyra, and one Prisma system). For the current report, T1-weighted (3D GRAPPA PAT 2, 1 mm^3^ isotropic, 256 × 256px, 192 slices, sagittal, ~5 min, TR 2500 ms, TE 4.33 ms, TI 110 ms, FA 7°) and T2-weighted (optimized for MTL volumetry, 0.5 × 0.5 × 1.5 mm^3^, 384 × 384px, 64 slices, orthogonal to the hippocampal long axis, ~12 min, TR 3500 ms, TE 353 ms) images were used. Standard operating procedures, quality assurance, and assessment were provided and supervised by the DZNE imaging network (iNET, Magdeburg) as described in [12].

### Volumetric analysis

#### Automated segmentation of hippocampal and parahippocampal subregions

Automated segmentation of hippocampal subfields (ASHS) was implemented in the entire DELCODE cohort using the Penn ABC-3T ASHS Atlas for T2-weighted MRI [9, 18, 19]. Using this atlas, hippocampal subfields (subiculum, dentate gyrus, Cornu Ammonis 1, 2, and 3, hippocampal tail) and parahippocampal regions (entorhinal cortex, Brodmann areas 35 and 36, parahippocampal cortex) were segmented in correspondence with the manual segmentation protocol by Berron et al. [9].

Each created segmentation mask underwent thorough quality assurance by an experienced rater. Quality ratings were performed separately for each hemisphere and for hippocampal and parahippocampal regions. In the present study, only participants whose segmentation masks passed the quality assurance for both hippocampal and parahippocampal regions in both hemispheres were included.

The quality assurance routine first included a visual inspection of all segmentation masks on five coronal and two sagittal snapshots. If there were no indications of segmentation errors, the respective mask was included for analyses. If it became apparent that segmentation failed, the respective segmentation mask was excluded from analyses. If the snapshots showed indications of possible segmentation errors, the respective segmentation mask was inspected in its entire three-dimensional extent. Here, any segmentation error that was visible on more than two consecutive slices on T2w MRI (i.e., extending more than 3 mm longitudinally) led to manual editing or exclusion of the segmentation mask. Errors affecting the outer boundaries of the segmented structures were edited in accordance with the manual segmentation rules by Berron et al. [9]. Errors that affected internal boundaries between subregions were not edited and led to exclusion of the respective segmentation mask. The rater was blinded to the diagnosis and amyloid status of the participants.

#### Total intracranial volume

Brain reserve may manifest already early in life [20]. While we sought to identify medial temporal lobe brain reserve, early-life manifestation of such reserve may be associated with widespread brain-effects, depending on the connectivity of the medial temporal region, and this may therefore affect total intracranial volume (TIV). Using TIV as a covariate would therefore weaken the ability to detect brain reserve enacted early in life. Therefore, we did not include TIV as a covariate in our analyses.

### Statistical analysis

We conducted a multivariate ANOVA (MANOVA) with segmentation volumes as dependent variables for regions of the hippocampal formation (four regions: CA1, CA3/CA2, DG, subiculum) and another one for parahippocampal regions (four regions: ErC, Brodmann areas 35 and 36, PhC) to assess the effect of diagnosis (asymptomatic versus SCD) and amyloid status (Aß42/40 ratio positive or negative), with age, sex, years of education, and site as covariates. Cook’s distances were used to detect outliers (> 0.6).

Paired comparisons were performed as post hoc paired comparisons on estimated marginal means (taking into account the same covariates) with Fisher’s LSD correction. These comparisons were limited to pair-wise comparisons of amyloid status within diagnostic groups.

An independent sample *t*-test was conducted to make two comparisons of four hippocampal subfields (CA1, CA2/3, DG, and subiculum): (1) between A+ asymptomatic and A+ SCD and (2) A− asymptomatic and A+ SCD individuals.

## Results

The sample characteristics are provided in Table 1. Volumes of hippocampal subfields and parahippocampal regions are reported in Tables 2 and 3, respectively. Planned, independent-sample *t*-tests showed that amyloid-positive (A+) individuals who were asymptomatic (stage 1) or had SCD (stage 2) in our sample did not differ with respect to years of education (*p* = 0.734), CSF total tau levels (*p* = 0.078), Aß42/40 levels (*p* = 0.257), MMSE scores (*p* = 0.947), or their PACC5 scores (*p* = 0.414). A+ SCD patients had significantly higher age (*p* = 0.023) and CSF phospho-tau levels (*p* = 0.03) than A+ asymptomatic individuals. Statistical comparisons between asymptomatic A− and SCD A− were not performed.

**Table 1:** Demographic data

Diagnosis	Asymptomatic			SCD
Aß42/40 status	−	+	−	+
*N*	95	26	104	47
Age	67.75 (4.76)	69.35 (5.28)	69.66 (5.9)	72.21 (4.94)
Sex (no. of females)	37	17	57	29
Years of education	14.42 (2.65)	14.54 (2.87)	15.08 (3.03)	14.79 (3.04)
Total tau	338.97 (127.25)	423.65 (158.77)	293.97 (101.92)	512.97 (224.9)
Phosphotau181	46.80 (13.43)	54.77 (18.58)	44.72 (14.57)	69.35 (30.5)
Aß42/40	0.106 (0.0134)	0.063 (0.014)	0.11 (0.013)	0.059 (0.012)
MMSE	29.4 (0.843)	29.23 (1.03)	29.09 (1.089)	29.21 (1.14)
PACC5	0.135 (0.514)	− 0.123 (0.801)	− 0.048 (0.626)	− 0.269 (0.683)
ApoE 4 carrier status	16%	56%	17%	61.4%

Mean values with standard deviations in parentheses. *MMSE* Mini-Mental State Exam. *PACC5* preclinical Alzheimer’s cognitive composite, version 5

**Table 2:** Volumes of hippocampal subfields

	Diagnosis	Abeta42/40 status	Mean	SD
**Dentate gyrus**	**Asymptomatic**	**−**	475.66	77.44
		**+**	505.40	89.28
	**SCD**	**−**	494.51	82.92
		**+**	450.93	74.19
**CA1**	**Asymptomatic**	**−**	691.88	90.88
		**+**	751.28	110.65
	**SCD**	**−**	719.44	119.15
		**+**	670.31	85.14
**CA3/CA2**	**Asymptomatic**	**−**	191.07	34.23
		**+**	206.34	37.53
	**SCD**	**−**	199.98	33.62
		**+**	184.86	27.79
**Subiculum**	**Asymptomatic**	**−**	1105.36	124.65
		**+**	1139.11	160.67
	**SCD**	**−**	1112.97	149.50
		**+**	1045.76	125.09

*SD* standard deviation

**Table 3:** Volumes of parahippocampal regions

	Diagnosis	Abeta42/40 status	Mean	SD
**Entorhinal cortex**	**Asymptomatic**	**−**	904.43	120.88
		**+**	923.02	117.94
	**SCD**	**−**	930.98	131.48
		**+**	846.99	101.18
**Brodmann area 35**	**Asymptomatic**	**−**	618.97	85.49
		**+**	648.97	97.66
	**SCD**	**−**	638.94	87.31
		**+**	638.47	88.75
**Brodmann area 36**	**Asymptomatic**	**−**	1929.73	300.41
		**+**	2092.95	427.32
	**SCD**	**−**	1984.20	345.57
		**+**	2044.23	367.07
**Parahippocampal cortex**	**Asymptomatic**	**−**	532.60	75.71
		**+**	555.78	75.54
	**SCD**	**−**	522.42	69.47
		**+**	508.71	71.30

*SD* standard deviation

### Comparison of volumes between groups

In a MANOVA for the hippocampal formation, there was no main effect of diagnosis (*F*(4261) = 0.759; *p* = 0.559) or amyloid status (*F*(4261) = 1.289; *p* = 0.275) but a significant interaction between diagnosis and amyloid status (*F*(4261) = 3.144; *p* = 0.015).

**Fig. 2:**
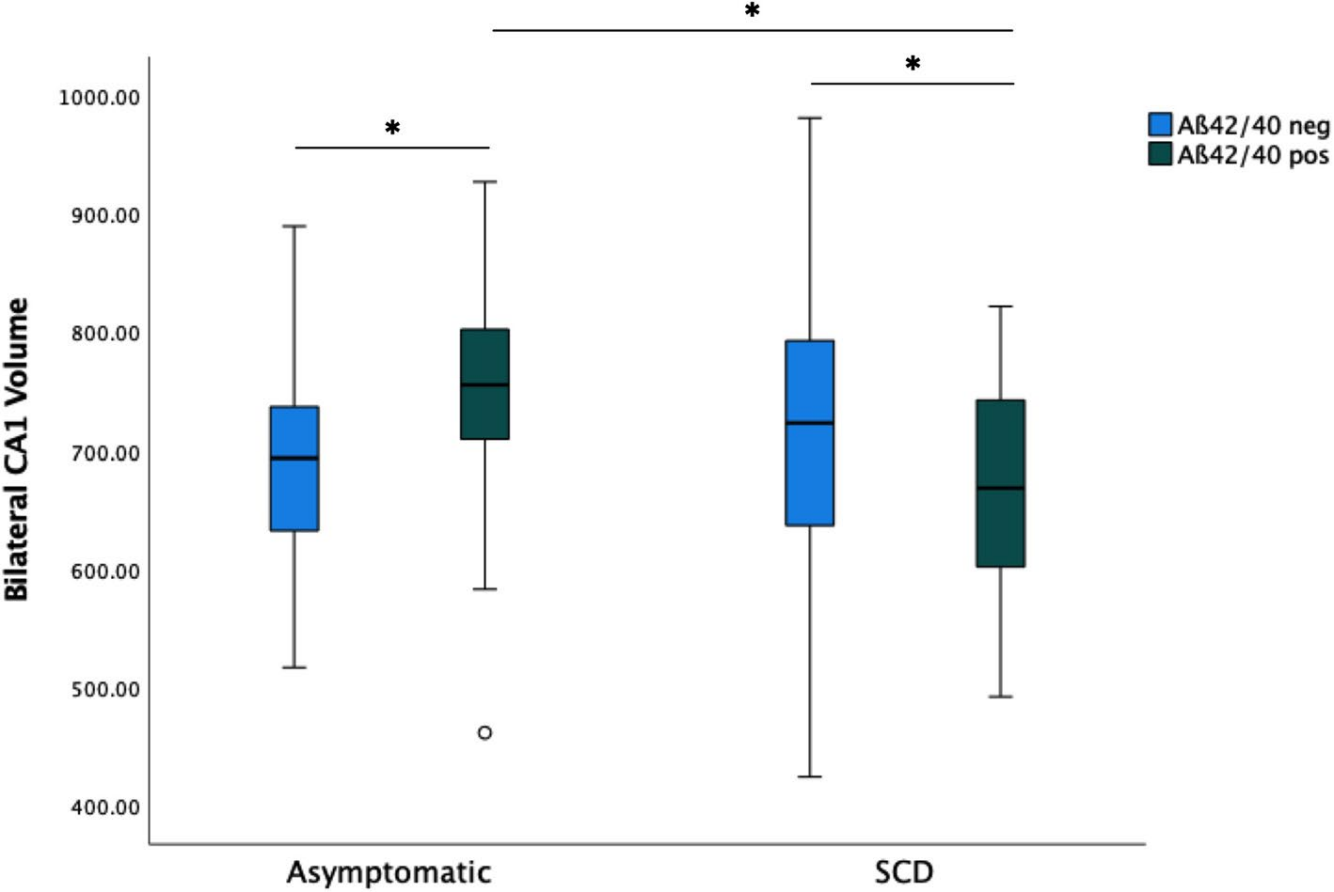
Bilateral volumes of the CA1 subfield (in mm^3^) in asymptomatic individuals and those with SCD. Aß42/40 positive asymptomatic individuals (stage 1) have larger CA1 subfields (*p* = 0.014) than asymptomatic and Aß42/40 neg. For SCDs, those that are amyloid-positive (stage 2) have smaller CA1 volumes than those that are amyloid-negative. Stage 1 individuals have larger CA1 volumes than stage 2 individuals (*p* < 0.001). Box and whisker plots show median (thick horizontal lines), minimum and maximum values (lower and upper end of whiskers), and outliers (circle, star). Whiskers below each box show the first quartile range and those above the fourth quartile range of data

Tests of between subject effects were significant for the interaction between diagnosis and amyloid status for the DG (*p* = 0.004), CA1 (*p* < 0.001), CA2/CA3 (*p* = 0.003), and the subiculum (*p* = 0.027). Analysis of Cook’s distances did not reveal any outliers (all values below 0.6). Post hoc pair-wise comparisons of estimated marginal means in the MANOVA (i.e., adjusted for the same covariates as in the MANOVA; Fisher’s LSD correction) showed significantly larger volumes in A+ than A− asymptomatic individuals for CA1 (*p* = 0.014) (Fig. 2) and CA2/CA3 (*p* = 0.047) and nonsignificant differences for DG (*p* = 0.175) and subiculum (*p* = 0.293). In SCD, lower volumes in A+ than in A− individuals were significant for DG (*p* = 0.004), CA1 (*p* = 0.025) (Fig. 2), CA2/CA3 (*p* = 0.026), and subiculum (*p* = 0.028).

We conducted an independent sample *t*-test to make two comparisons of four hippocampal subfields (CA1, CA2/3, DG, and subiculum): (1) between A+ asymptomatic and A+ SCD and (2) A− asymptomatic and A+ SCD. Results revealed that A+ asymptomatic individuals had larger CA1 (*p* < 0.001), CA2/CA3 (*p* = 0.006), DG (*p* = 0.007), and subiculum (*p* = 0.004) volumes in the first comparison. Although A+ SCD individuals showed smaller subfields than A− asymptomatic individuals in the second comparison, this did not reach the significance level for CA1 (*p* = 0.168), CA2/3 (*p* = 0.282), and DG (*p* = 0.072) except subiculum (*p* = 0.008) (see Fig. 2).

For parahippocampal regions, the main effect of diagnosis was not significant (*F*(4260)=2.278; *p* = 0.661), the main effect of amyloid status was not significant (*F*(4260)=3.376; *p* = 0.845), and their interaction was not significant (*F*(4260)=2.111; *p* = 0.623). Test of between subject effects and post hoc pair-wise comparisons were not further considered given the lack of significant main-effects and interactions.

## Discussion

We found an interaction between hippocampal subfield volumes and amyloid status in asymptomatic individuals and SCDs (Fig. 2). This interaction is in agreement with the hypothesis that stronger brain reserve, as indicated by brain volumes contributes to distinguishing the stage 1 from stage 2 of the Alzheimer’s continuum. The brain reserve interpretation is supported by the larger CA1 subfield (*p* = 0.014) and larger CA2/CA3 subfields (*p* = 0.047) in asymptomatic A+ subjects in a post hoc pair-wise comparison with asymptomatic A− participants (Fig. 2). These findings indicate a higher than expected brain integrity as expressed by a larger CA1 and CA2/CA3 volumes in the presence of amyloid pathology in stage 1 individuals, which is in agreement with the recent Reserve and Resilience working group framework definitions for brain reserve [5].

We found that the interaction between amyloid status and clinical status was particularly strong in the subfields DG, CA1, CA2/CA3, and subiculum, and these subfields were smaller in A+ and A− SCD. Memory processing in the subfields of the hippocampal formation and parahippocampal subregions is organized along designated circuits. The DG supports “pattern separation” of similar events, while CA3 and CA1 enable pattern completion and associative learning, respectively. CA1 also allows orchestrated cortical reinstatement of mnemonic information and novelty detection [21–24]. The subiculum, in turn, is a major output structure of the hippocampus with a widespread connectivity including other regions of the episodic memory network, such as the retrosplenial region [25, 26]. Therefore, in this scheme, our findings suggest that particularly aspects related to pattern separation, novelty processing, and associative learning should contribute to brain reserve in stage 1. In contrast, the unitization of information and familiarity-based recognition seems to involve the adjacent perirhinal cortex [27–30]. Perirhinal cortex and adjacent regions (the entorhinal cortex) did not show a significant interaction between amyloid status and clinical stage, suggesting that unitization and familiarity-recognition may not contribute to brain reserve in stage 1.

While the A+ SCDs had numerically smaller volumes than the asymptomatic A− group (Table 2) in all subfields, this difference was only significant in the subiculum. These results suggest that stage 2 is associated with only a subtle atrophy when compared to amyloid-negative older adults without memory complaints. One possible interpretation of these results is that patients with A+ SCD are those individuals who were initially the asymptomatic A− group and developed memory complaints under amyloid pathology through a combination of subtle atrophy (significant in the subiculum) and synaptic dysfunction associated with amyloid oligomers. To what extent the atrophy in the subiculum may play a specific role in contributing to memory complaints in stage 2 remains to be determined and replicated.

Years of education was not different between asymptomatic A+ and A− or between asymptomatic A+ and SCD A+. Hence, brain reserve in stage 1 does not appear to be related to higher levels of education. The neurobiological underpinning of this reserve remains to be determined. Candidate mechanisms may include polygenic factors [31]. There is also the possibility that stage 1 and stage 2 are distinguished by fewer ApoE4 carriers in stage 1 (Table 1). However, our study was not sufficiently powered to assess a three-way interaction between amyloid status, clinical stage, and ApoE4 status.

This study has a number of strengths. We recruited stage 2 in a health care-based approach and therefore our data directly speak to brain reserve in cognitively normal individuals who do not seek medical advice due to memory complaints. We used a new and accurate segmentation protocol that allows accurate segmentation of hippocampal subfields and parahippocampal regions. Finally, we performed a stringent QA of the segmentation results.

### Limitations

There are also a short-comings. The sample size was small particularly for the stage 1 individuals and therefore our findings need replication. Furthermore, we did not have a sufficient sample size to stratify according to ApoE4 status.

## Conclusion

We provide first evidence that large volumes of hippocampal subfields, particularly CA1, could present a brain reserve mechanism that allows individuals with amyloid-pathologic change to be cognitively normal without experiencing subjective cognitive decline. This effect is not predicted by the level of education. While these findings require replication, they have implications for preclinical AD trials and disease-modifying treatments in preclinical individuals.

## Data Availability

The data, which support this study, are not publically available but may be provided upon reasonable request.

## Abbreviations

A+: Amyloid-positive
A−: Amyloid-negative
AD: Alzheimer’s disease
ASHS: Automatic segmentation of hippocampal subfields
CA: Cornu Ammonis
CSF: Cerebrospinal fluid
DELCODE: DZNE-Longitudinal Cognitive Impairment and Dementia Study
DG: Dentate gyrus
DSCT: Digit-Symbol Coding Test
DSI: Dice similarity index
DZNE: German Center for Neurodegenerative Diseases
FCSRT: Free and Cued Selective Reminding Test
HCs: Healthy controls
ICC: Intraclass correlation coefficients
MCI: Mild cognitive impairment
MMSE: Mini-Mental State Examination
MRI: Magnetic resonance imaging
PhC: Parahippocampal cortex
SCD: Subjective cognitive decline
SD: Standard deviation
TIV: Total intracranial volume
QA: Quality assessment
